# Sensitive Commercial NASBA Assay for the Detection of Respiratory Syncytial Virus in Clinical Specimen

**DOI:** 10.1371/journal.pone.0001357

**Published:** 2007-12-26

**Authors:** Ramona Liza Tillmann, Arne Simon, Andreas Müller, Oliver Schildgen

**Affiliations:** 1 University of Bonn, Institute for Virology, Bonn, Germany; 2 University of Bonn, Children's Hospital, Bonn, Germany; Medical University of South Carolina, United States of America

## Abstract

The aim of the study was to evaluate the usability of three diagnostic procedures for the detection of respiratory syncytial virus in clinical samples. Therefore, the FDA cleared CE marked NOW® RSV ELISA, the NucliSENS® EasyQ RSV A+B NASBA, and a literature based inhouse RT-PCR protocol were compared for their relative sensitivities. Thereby, NASBA turned out to be the most sensitive method with a total number of 80 RSV positive samples out of a cohort of 251 nasopharyngeal washings from patients suffering from clinical symptoms, followed by the inhouse RT-PCR (62/251) and ELISA (52/251). Thus, NASBA may serve as a rapid and highly sensitive alternative for RSV diagnostics.

## Introduction

Despite an increasing number of newly detected respiratory pathogen the human Respiratory syncytial virus (RSV) remains the single most prevalent etiologic agent in pediatric viral respiratory tract infection [1], [2], [3]. RSV is responsible for the majority of episodes of acute wheezing triggered by infection [4], bronchiolitis [5] and pneumonia [6] predominantly during the first 24 months of life. An estimated percentage of about 1–2% of all RSV-infected children require hospital care. The RSV-related hospitalization rate and the risk of severe complications are increased in prematurely born infants with chronic lung disease (CLD) [7] and in children with hemodynamically relevant congenital heart disease (CHD) [8], [9], other forms of chronic lung disease or severe neuromuscular impairment [2]. Forster and coworkers estimated (95% confidence interval) a total of 26,524 (23,812–29,432) RSV-related hospitalizations per year in children under 3 years of age in Germany (i.e. 38% of all pediatric hospitalizations for viral lower respiratory tract infection) [10]. The same group calculated €2.772 as median total costs per hospitalised RSV-Infection [11], [12], [13]. Others recently calculated even higher costs [14]. Specific therapeutic agents with proven efficacy against RSV are still not available [1], [5]. Meticulous hand hygiene after patient contact together with other barrier precautions and rapid laboratory diagnostic are considered to be of utmost importance for the prevention of nosocomial transmission [16], [17]. Rapid laboratory detection of RSV is mainly performed by ELISA [17], [18] or by the use of nucleic acid amplification and detection methods. The latter methodology includes a high number of RT-PCR protocols, but for reasons of quality assurance in quality management systems the need for standardized nucleic acid amplification procedures with quality marks like the CE mark increases more and more. Recently, the NucliSENS EasyQ RSV A/B (bioMerieux, Nürtingen, Germany), a CE-labeled Nucleic acid sequence based amplification (NASBA) based kit for the rapid detection of RSV, became available. In search for options to optimize the rapid laboratory diagnostics of RSV we have compared this NASBA method with a published RT-PCR protocol and a rapid ELISA, the latter both used in our routine procedures for the detection of RSV.

## Materials and Methods

The patient cohort consisted of a total number of 251 pediatric patients hospitalized with respiratory tract infection. Only one clinical sample per patient was included in the study, resulting in a total number of 251 nasopharyngeal aspirates. These aspirates were used freshly for all subsequent procedures and were not frozen before usage. All specimens were previously tested negative by PCR or RT-PCR as previously described [16], [2] for any of the following viruses: human bocavirus, human metapneumovirus, Influenzaviruses A and B, and human coronaviruses NL63, HKU1, SARS, OC43, and 229E. Additional tests to detect Rhinoviruses, Adenoviruses, Parainfluenzaviruses, or bacteria, were not performed in our laboratory. The main focus of the present study was to evaluate the sensitivity and specificity of the NASBA method and the rapid ELISA compared to RT-PCR.

Native samples were tested by the FDA cleared CE marked NOW® RSV ELISA (Inverness Medical, Cologne, Germany). NOW® RSV ELISA tests were carried out strictly following the manufacturer's protocol and considered positive according to the manufacturer's guidelines. For NSABA and RT-PCR RNA was automatically extracted by the NucliSENS® easyMAG™ (bioMerieux, Nürtingen, Germany) using the manufacturer's extraction protocol for nasopharyngeal specimen, using 100 µl of specimen preincubated for 30 min at 37°C with 10 µl DNase and 12 µl DNase buffer (Promega, Germany). Subsequent NASBA reactions were carried out using the NucliSENS® EasyQ (bioMerieux, Nürtingen, Germany) system strictly following the manufacturer's guidelines. RNA used for RT-PCR was extracted as described above. RT-PCR was performed essentially as previously described by Mentel and coworkers [19]. Briefly, reverse transcription was carried out with the Expand Reverse Transcriptase (Roche, Mannheim, Germany) for 30 min at 42°C with primers F1 Forward primer GTTGGATCTGCAATCGCCAGTGGC and F2 Reverse primer GTACATAGAGGGGATGTGTG, followed by a five minute denaturation step at 99°C. First round PCR was performed with primers F1 and F2 (1×5′94°C; 40×30″94°C, 30″54°C, 45″72°C; 1×5′72°C) using the Expand High Fidelity PCR system (Roche, Mannheim, Germany) according the producer's recommendation. The second round of PCR was performed with the identical temperature profile but with nested primers F3 Forward primer TTAACCAGCAAAGTGTTAGA and F4 Reverse primer TTTGTTATAGGCATATCATTG.

## Results and Discussion

The results are summarized in table 1. From the 251 specimen 52 (20.7%) were tested positive for RSV by NOW® RSV ELISA (Inverness Medical, Cologne, Germany), 62 (24.7%) were tested positive for RSV by RT-PCR, and 80 (31.9%) were tested positive for RSV by NucliSENS® EasyQ NASBA (bioMerieux, Nürtingen, Germany). Thus, as the highest sensitivity was observed for the CE marked NucliSENS® EasyQ, this relative sensitivity was set to 100%. Thereby it was assumed that with NucliSENS® EasyQ according to the CE marked guarantee a sensitivity and specificity of this test of 99% as earlier described [20]. In relation to the positive test results obtained with the NucliSENS® EasyQ NASBA, the relative sensitivity of the RT-PCR was 77,5% compared to 65% obtained with the NOW® RSV ELISA.

**Table 1:** Overview on the comparison of three diagnostic procedures for the detection of RSV

	ELISA	RT-PCR	NASBA	ALL	RT-PCR/NASBA	RT-PCR/ELISA	NASBA/ELISA	RT-PCR only	NASBA only	ELISA only
No. of positive specimen	52	62	80	43	16	4	0	1	17	4
Percent from total cohort	20.72%	24.70%	31.87%	17.13%	6.37%	1.59%	0%	0.4%	6.77%	1.59%
Relative sensitivity	65%	77.5%	100%							

A total number of 43 (17.1%) samples were tested positive for RSV by all methods, 16 samples (6.37%) were tested positive for RSV by both RT-PCR and ELISA but not by ELISA, 4 samples (1.59%) were tested RSV positive by RT-PCR and ELISA but not by NASBA, 1 sample (0.4%) was tested RSV positive only by RT-PCR, 4 samples (1.59%) were tested RSV positive only by ELISA, and 17 samples (6.77%) were tested RSV positive only by NASBA. The overall accordance of all techniques was 53.75%, the accordance of RT-PCR and NASBA was 76.25%, the accordance of NASBA and ELISA was 60%, and the accordance of RT-PCR and ELISA was 78.69%. The negative predictive values for were 97.1% for NASBA, 83.4% for ELISA, and 87.8% RT-PCR, the positive predictive values were 94.1% for NASBA, 92.9% for ELISA, and 100% RT-PCR (table 2). The results showed that from the three tested methods for molecular diagnosis of RSV the NucliSENS® EasyQ NASBA (bioMerieux, Nürtingen, Germany) detected the most RSV positive samples in a cohort of 251 nasopharyngeal samples of pediatric patients hospitalized with respiratory disease.

**Table 2:** Overview on the predictive values of three diagnostic procedures for the detection of RSV

Method	Negative Predictive Value	Positive Predictive Value
NASBA	97.1%	94.1%
ELISA	83.4%	92.9%
RT-PCR	87.8%	100%

Taking into account data published in the manufacturer's manuals and on their respective websites, it can be assumed that the specificity of both NucliSENS® EasyQ NASBA and the NOW® RSV ELISA is very high. Furthermore, as demonstrated last year by Manji and coworkers, the NucliSENS® EasyQ NASBA assay specifity has a positive sample value of ≥1.100 with an acceptable IC value of ≥1.100 [21]. Thereby, the absolute assay specifity turned out to be ≥95%. For the NOW® RSV ELISA Cruz et al. [22] determined that the sensitivity was 81% and specificity 93.2%. Moreover, with our in house RT-PCR we have not yet any false positive as all detections were confirmed by sequencing (Simon, Schildgen *et al*., unpublished data). It is commonly known that antibody based methods ELISA like for detection of RSV in clinical samples is less sensitive than nucleic acid amplification techniques [23]. However, the rapid results are of high importance for clinicians in order to initiate therapy and/or isolation of the patients in order to avoid nosocomial outbreaks. In this earlier study which solely compared rapid ELISA methods, the NOW® RSV ELISA was found to be the most sensitive at least for the cohort of pediatric patients [17] with hands on time of about 10 to 20 min. However, taken into account the higher relative sensitivity and the acceptable predictive values accompanied by short hands on time and final results in nearly of 90 min, the NucliSENS® EasyQ NASBA may serve alternative method as it is both a fast but also a highly sensitive method. It thus should be taken into account whenever rapid and sensitive RSV diagnostics are required, such as in clinical setting involving high risk patients for which nosocomial outbreaks may be a fatal event.
